# Characterization of the complete chloroplast genome of *Homalomena occulta* (Lour.) Schott

**DOI:** 10.1080/23802359.2021.1895690

**Published:** 2021-03-18

**Authors:** Ke Zhang, Kongju Wu, Suxiang Lu, Junfeng Zhang, Xiaodong Ma, Yanzi Wang, Li Huang, Xiangting Wang, Xichao Xia

**Affiliations:** a College of Medicine, Pingdingshan University, Pingdingshan City, Henan Province, China; b Henan Province Key Laboratory of Germplasm Innovation and Utilization of Eco-economic Woody Plant, Pingdingshan City, Henan Province, China

**Keywords:** *Homalomena occulta* (Lour.) Schott, chloroplast genome, Illumina sequencing

## Abstract

*Homalomena occulta* (Lour.) Schott (*H. occulta*) is a traditional Chinese medicine. However, the chloroplast genome has not been reported. Here, we assembled and analyzed the complete chloroplast (CP) genome of *H. occulta.* We found that the *CP* genome of *H. occulta* is 165,398 bp in length and contains a large single-copy (LSC) region of 92,861 bp, a small single-copy (SSC) region of 20,943 bp and an inverted repeat (IR) region of 25,797 bp. The genome contains 130 genes including 85 protein-coding genes, 8 rRNA and 37 tRNA. Phylogenetic analysis indicated that *H. occulta* is close to *Philodendron lanceolatum.* This study provides useful data for the development of molecular markers and identification of *H. occulta.*

*Homalomena occulta* (Lour.) Schott (*H. occulta*) (Family: *Araceae*) is mostly found in the Guangxi and Yunan Provinces in China and is used as a traditional Chinese medicine. *Homalomena occulta* has several medicinal properties and is used as an anti-inflammatory and analgesic. It can be used for the treatment of cold and wet arthralgia, cold pain of the waist and knee and numbness of the lower extremity contracture (Xie et al. 2012; Yang et al. 2019).

The chloroplast genome plays an important role in species identification; however, the chloroplast genome has not yet been reported. In this study, we sequenced the complete chloroplast genome of *H. occulta* collected from the South China Garden of Botany of Guangdong Province China (E113.3649, N23.1825) stored in the herbarium of Pingdingshan University (Specimen code PDS20191016). This species was identified using the Illumina Novaseq 6000 platform with a genomic shotgun library with an insertion size of 350 bp (Raw data 3.81 G). The clean data (3.77 G) were assembled *de novo* using the SPAdes v. 3.11.0 software (Bankevich et al. 2012). The assembled complete chloroplast genome was annotated using the Plann software (Huang and Cronk 2015) and submitted to GenBank under the accession number MW145396.

The total length of the chloroplast genome of *H. occulta* is 165,398 bp, with a GC content of 35.7%. Similar to other chloroplast genomes, the chloroplast genome of *H. occulta* has a quadripartite structure including a large single-copy (LSC) region of 92,861 bp, a small single-copy (SSC) region of 20,943 bp and an inverted repeat (IR) region of 25,797 bp. The complete chloroplast genome of *H. occulta* contains 130 genes including 85 protein-coding genes, 8 rRNA and 37 tRNA. The *clp*P and *ycf*3 genes contain two introns, respectively. The *rps*12 gene has one trans-splicing site.

To determine the phylogenetic position of *H. occulta*, 76 protein-coding genes of the chloroplast genome of *H. occulta* were aligned with that of 8 other species in GenBank using the HomBlocks software (Biet al. 2018). The phylogenetic tree was constructed using the maximum likelihood method in the RAxMLv8.2.9 software (Stamatakis 2014). Our results indicated that *H. occulta* is closely related to *Philodendron lanceolatum* (Figure 1). Our results provide useful data for the development of molecular markers and identification of *H. occulta.*

**Figure 1.**
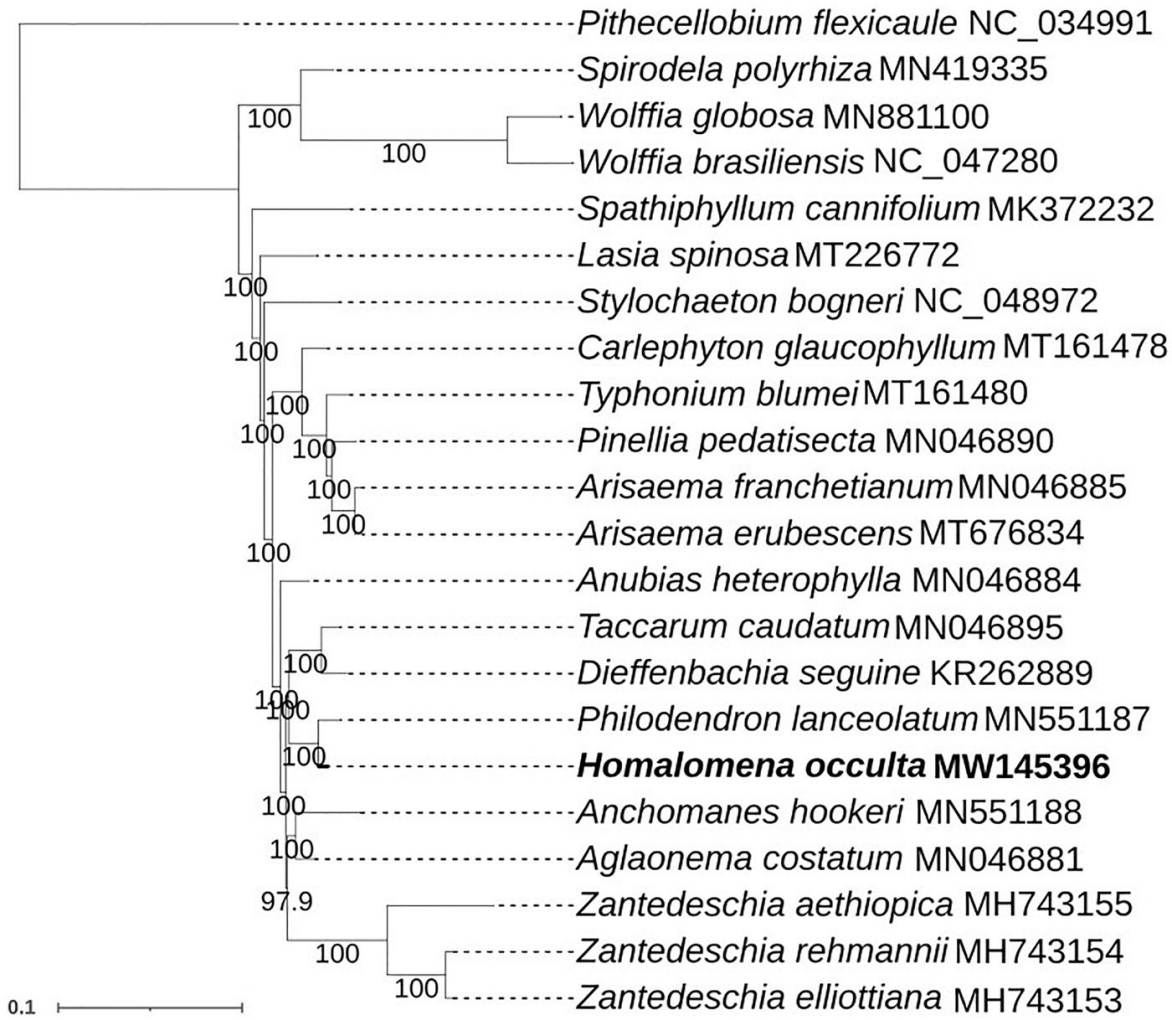
Phylogenetic relationships of H. occulta inferred from nucleotides of 75 PCGs maximum-likelihood (ML) methods. Sequences were blast by using Mafft v7.037, ML tree was constructed by using MAGE 7 and set bootstrap 1000. Pithecellobium flexicaule (NC_034991) was set as the outgroup.

## Data Availability

The complete chloroplast genome of *H. occulta* that was generated in this study was submitted to GenBank under the accession MW145396 number (https://www.ncbi.nlm.nih.gov/nuccore/MW145396) and SRA data was submitted under the accession number PRJNA675187.
